# Green Synthesis of Zinc Oxide Nanoparticles for Enhanced Adsorption of Lead Ions from Aqueous Solutions: Equilibrium, Kinetic and Thermodynamic Studies

**DOI:** 10.3390/molecules22060831

**Published:** 2017-06-08

**Authors:** Susan Azizi, Mahnaz Mahdavi Shahri, Rosfarizan Mohamad

**Affiliations:** 1 Department of Bioprocess Technology, Faculty of Biotechnology and Biomolecular Sciences, Universiti Putra Malaysia, UPM Serdang, Selangor 43400, Malaysia; susanazizi@yahoo.com; 2 Department of Chemistry, Shiraz Branch, Islamic Azad University, Shiraz 74731-71987, Iran; 3 Laboratory of Biopolymer and Derivatives, Institute of Tropical Forestry and Forest Products, Universiti Putra Malaysia, UPM Serdang, Selangor 43400, Malaysia

**Keywords:** ZnO nanoparticles, heavy metals, adsorption, green chemistry

## Abstract

In the present study, ZnO nanoparticles (NPs) were synthesized in zerumbone solution by a green approach and appraised for their ability to absorb Pb(II) ions from aqueous solution. The formation of as-synthesized NPs was established by X-ray diffraction (XRD), Transmission Electron Microscopy (TEM), and UV–visible studies. The XRD and TEM analyses revealed high purity and wurtzite hexagonal structure of ZnO NPs with a mean size of 10.01 ± 2.6 nm. Batch experiments were performed to investigate the impact of process parameters viz. Pb(II) concentration, pH of solution, adsorbent mass, solution temperature, and contact time variations on the removal efficiency of Pb(II). The adsorption isotherm data provided that the adsorption process was mainly monolayer on ZnO NPs. The adsorption process follows pseudo-second-order reaction kinetic. The maximum removal efficiencies were 93% at pH 5. Thermodynamic parameters such as enthalpy change (ΔH^0^), free energy change (ΔG^0^), and entropy change (ΔS^0^) were calculated; the adsorption process was spontaneous and endothermic. The good efficiency of the as-synthesized NPs makes them attractive for applications in water treatment, for removal of heavy metals from aqueous system.

## 1. Introduction

The removal of heavy metals from water and wastewater is a matter of concern worldwide. These heavy metals are of serious health and environmental concern and there is a need to discover new and effective methods for their removal from industrial effluents [1]. Heavy metals reach tissues through the food chain and accumulate in the human body. If the metals are ingested beyond the permitted concentration, they can cause serious health disorders [2]. Lead ion is one of the heavy metals considered toxic to humans and aquatic life when present in high quantities in water. The presence of lead ions in drinking water above the acceptable limit (5 ng/mL) may cause harmful health effects such as anaemia, encephalopathy, hepatitis, nephritic syndrome. Various approaches have been developed for the removal of heavy-metal ions from water/wastewater which include chemical precipitation/coagulation, membrane technology, electrolytic reduction, ion exchange and adsorption. Adsorption is one of the most common techniques used to removal heavy-metal ions due to its simplicity and high efficiency, as well as the ease of use of a wide range of adsorbents. Numerous studies have explored various nanoparticles for adsorption of heavy metals, owing to the simplicity of modifying their surface functionality and their high surface area to volume ratio for enhanced adsorption capacity and efficiency [3]. Nanosized metal oxides, including nanosized manganese oxides, ferric oxides, aluminum oxides, magnesium oxides and cerium oxides are considered as the capable ones for heavy metals adsorption from aqueous systems [4]. To date, these nanomaterials are extensively explored as highly efficient absorbents for heavy metal ions removal from water/wastewater. They display some advantages such as high ability, unsaturated surfaces, simple operation, rapid kinetics, and desirable sorption toward heavy metals in water and wastewater [5]. As a low-toxicity material, ZnO has various applications in different areas including catalyst industry [6], gas sensors [7] solar cells [8] and medicine [9]. As an adsorbent, ZnO proved to be the most effective than other absorbents such as phosphate, iron oxide, and activated carbon for sulfur compounds removal and H_2_S, due to more favorable sulfidation thermodynamics [10]. Recently in some literatures have been reported that ZnO NPs could efficiently absorb heavy metals from aqueous systems [11].

Use of plants extracts in synthesis of nanoparticles is relatively novel leading to accurately green chemistry as it is eco-friendly, low cost and smoothly scaled up for large scale production [12,13]. In addition, the biomolecules of plant extract will chemically bind to the surface of the nano-structures, stabilize the nanoparticles and prevent their aggregation as well leads to arising consequent surface effects during their application [14]. In this paper, we are the first to report the green synthesis of ZnO-NPs using zerumbone extract. Zerumbone is a monocyclic sesquiterpenoid which can be found plentifully in rhizomes mostly from *Zingiber zerumbet Smith* and *Zingiber aromaticum* [15,16]. Additionally, the potential use of green synthesized ZnO NP as a nano-adsorbent (ZnO-Nano-A) in the removal of Pb(II) by determining the maximum adsorption capacity was investigated. Langmuir and Freundlich adsorption isotherm models were applied to fit the equilibrium isotherm. The adsorption kinetics models and the thermodynamics of adsorption for Pb(II) ions were also evaluated.

## 2. Results and Discussion

### 2.1. Characterization of ZnO NPs

**Figure 1:**
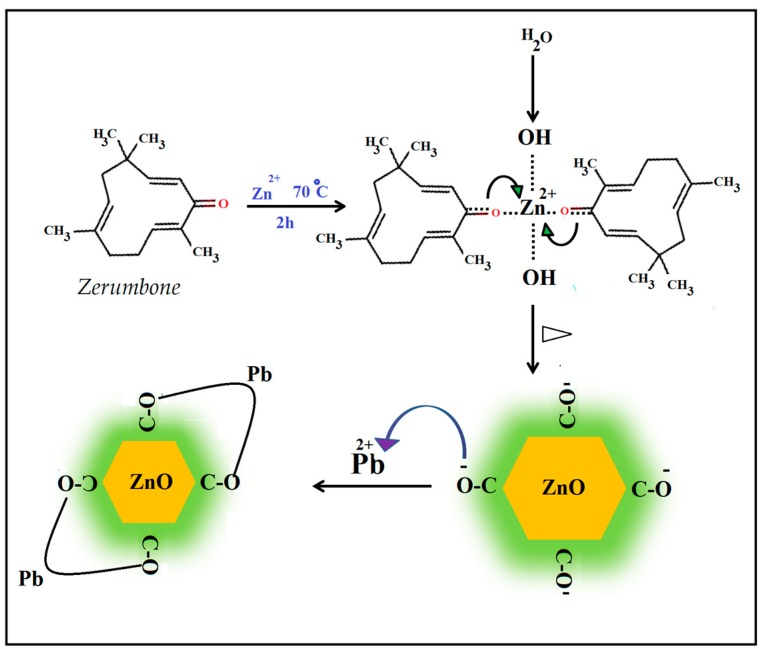
Schematic illustration of the synthesis and functionalization of the zerumbone-stabilized ZnO NPs, and possible chelating of Pb^2+^ ions to zerumbone.

Formation of ZnO NPs was visually evident from the color change of reaction mixture from colorless to white after 30 min of reaction. The presence of carbonyl groups and extensive number of π electrons in the zerumbone molecular structure can enable the complexation of zinc cations (Zn^2+^) to molecules of zerumbone followed by hydrolysis, and finally formation of ZnO NPs through thermal decomposition of Zn(OH)_2_ complex as a unique source precursor. This structure helps zerumbone to stabilize zinc particles and eventually ZnO NPs while preventing their extreme aggregation or crystal growth In addition, the stabilizing of ZnO NPs by zerumbone, which acts as donor of electrons, leads to formation of active adsorption sites to absorb heavy metal ions from aqueous system (Figure 1).

**Figure 2:**
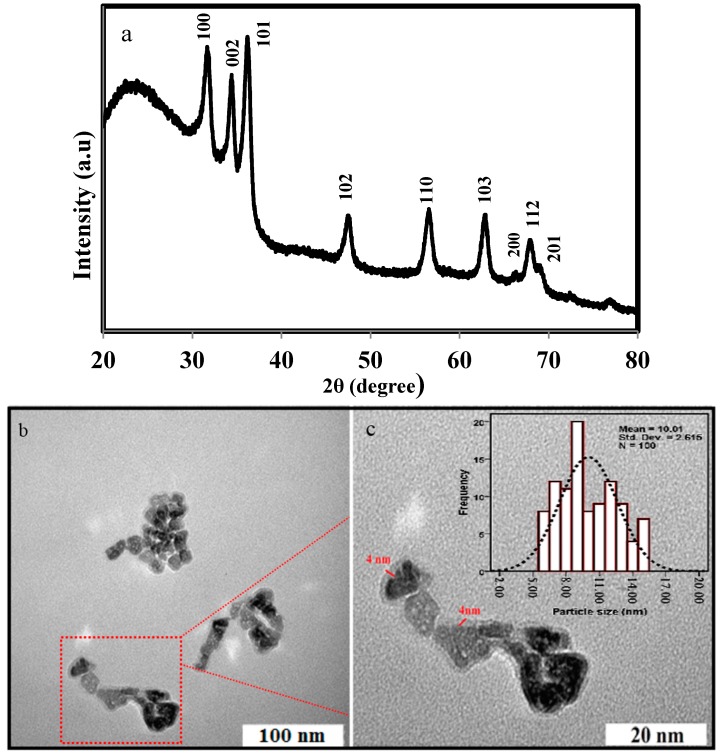
(**a**) The XRD pattern; (**b**,**c**) TEM images and (inset) particle size distribution histogram of ZnO-NPs.

The XRD pattern of the bioformed ZnO-NPs is shown in Figure 2a. The XRD pattern shows the all of the characteristics peaks of ZnO-NPs with Miller indices (100), (002), (101), (102), (110), (103), (200), (112), and (201) which can be indexed to reflections of the ZnO wurtzite structure (JCPDS 36-1451). The presence of zerumbone on the surface of nanoparticles was confirmed with a halo of the typical peak at 2θ of 22.93°. Line broadening and sharpness of the diffraction peaks are evidences respectively, which the as-synthesized particles are in the nanometer range and crystals. The crystal size of the ZnO NPs was calculated using Debye-Scherrer equation which was around 10 nm. The morphology and structure of the ZnO NPs were investigated by transmission electron microscopy (TEM). The particle size and size distribution of ZnO NPs were calculated by measuring the diameters of around 100 nanoparticles chosen randomly through the TEM images. Figure 2b,c show typical TEM images of bioformed ZnO NPs in two different magnifications. The micrograph 2b shows the polydispersed nanostructures with a regular hexagonal surface shape which are in agreement with the XRD result.

At higher magnification micrograph (Figure 2c), the well-defined surface modification with a thickness of ~4 nm, revealing the presence of coating, was obvious. The results are consistent with the concept that the ZnO nanoparticles are firmly coated with zerumbone and this process inhibits further aggregation or agglomeration between the final nanoparticles. Particle size distribution histogram revealed an average particle diameter of ZnO of 10.01 ± 2.6 nm (inset Figure 2c), which is well matched with the measured crystal diameter obtained from XRD result.

**Figure 3:**
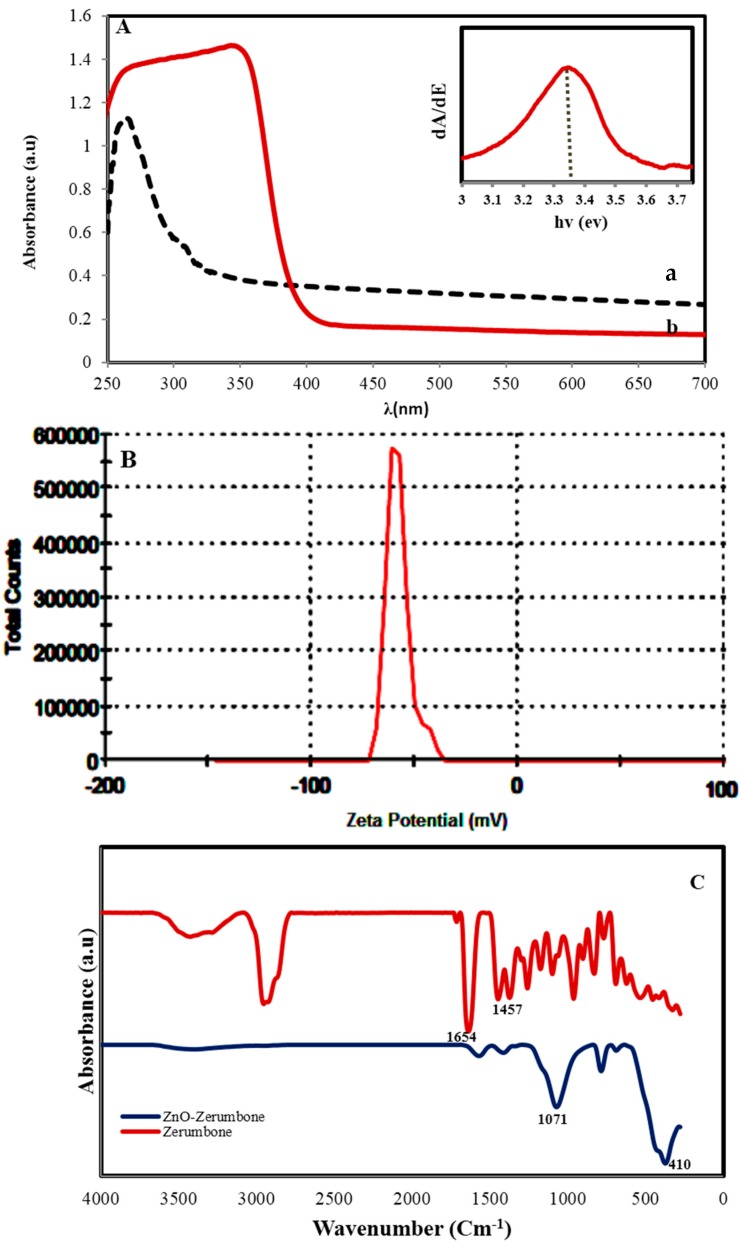
(**A**) UV–Vis spectrum ((a) zerumbone (b) bio-synthesized ZnO NPs, and (inset) band gap estimation) (**B**) zeta potential of ZnO NPs and (**C**) the FTIR spectrum of pure zerumbone before and after biosynthesis of ZnO NPs.

The UV–vis absorption spectrum (Figure 3A) shows a typical absorption peak of ZnO at a wavelength of 353 nm which can be ascribed to the band-gap absorption of ZnO due to the electron transitions from the valence band to the conduction band (O2p-Zn3d) [17]. Furthermore, this peak shows that the particles are nanoscale, with a narrow particle size distribution. The absorption peak of zerumbone at 250 nm, which is related to the π→π * transitions in the sesquiterpene system can hardly be seen in the UV spectrum of ZnO NPs, may be due to change in the sesquiterpene structure and absence of this π→π * transitions. The band gap of ZnO NPs was obtained through the first derivative of the absorbance in regard to photon energy and the maximum in the derivative band was seen at the lower energy edges. The derivative of the absorbance of the ZnO NPs is shown in inset figure and it illustrates a band gap of 3.34 eV for the ZnO NPs. Zeta potential (ZP) value (Figure 3B) displays information about the surface charge and stability of bioformed ZnO NPs. The average ZP value of −57.70 mV specified that the surfaces of ZnO NPs are coated with molecules which are mainly included of negatively charged groups and similarly responsible for stability of the nanoparticles [18]. This result showed nanoparticles have considerable active adsorption sites to absorb heavy metal ions from aqueous systems. ICP-AES analysis of Zn^2+^ content after formation of ZnO by extract was found to be 185 ppm at pH 7. The percent yield (%Y) was calculated as 92%.

The FTIR spectrum of pure zerumbone before and after biosynthesis of ZnO NPs is shown in Figure 3C. The characteristic FTIR peaks of zerumbone alone over the range 500–4000 cm^−1^ appear at 1654 cm^−1^ (α,β-unsaturated ketone and ethylenic bands) [19]. The peak at 1457 cm^−1^ is due to the C=C groups and the peaks below 1000 cm^−1^ resulted from C–H bending. The peak close 2935 cm^−1^ indicates a CH_2_ stretching vibration and the broad peak at 3465 cm^−1^ is due to an OH stretching vibration probably originating from the residual alcohol. After synthesis of ZnO NPs important differences in the intensity, shape and position of peaks indicate the contribution of the functional groups of zerumbone to the formation and coating of nanoparticles. Most of the peaks below 1750 cm^−1^ disappeared, shrank and shifted, indicating the C=O and C=C groups participated in the production and coating of nanoparticles. In fact the π electrons of C=O and C=C groups can transfer to the free orbital of Zn^2+^ ions. Such interactions would decrease the mobility of ions and after formation of ZnO NPs inhibit the growth of large particles. The appearance of a strong band at 1071 cm^−1^ attributed to C–O vibration is an evidence for the binding of C–O groups to the surface of the nanoparticles, which this surface structure can contribute for the absorption of metal ions. These observations are in consistent with the UV-Vis absorption result. The formation of ZnO NPs is clearly confirmed by an intense band at 410 cm^−1^.

### 2.2. Evaluation of Adsorption Mechanism

#### 2.2.1. Effect of Contact Time

**Figure 4:**
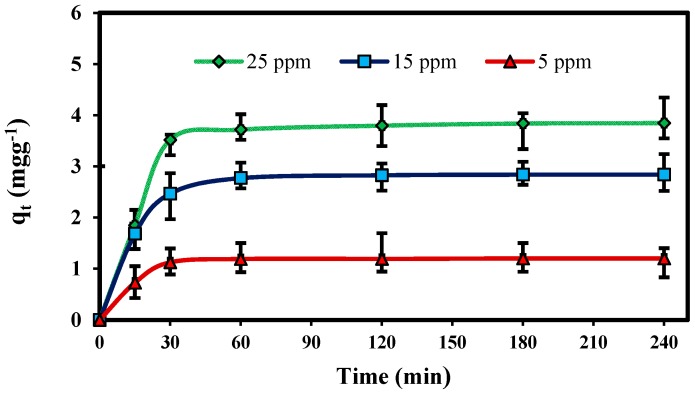
Effect of contact time on the adsorption of Pb(II) onto ZnO-Nano-A at different concentration: (adsorbent mass, 0.1 g L^−1^; initial pH, 5.0; solution volume, 25 mL; temperature, 30 °C).

The kinetic study for adsorption of Pb(II) ions in aqueous solution at different concentration 5, 15 and 25 mg L^−1^ at 30 °C with fixed adsorbent mass is shown in Figure 4. The results show that the adsorption rate initially increased rapidly with increasing contact time, and then adsorption equilibrium was reached after 60 min for Pb(II) ions. In addition, the adsorption capacity achieved a constant value after equilibrium had been reached. This probably resulted from saturation of nano-adsorbent surfaces with metal ions followed by adsorption and desorption processes that occur after saturation. The results indicate that the adsorption capacity of Pb(II) increased from 1.26 to 3.82 mg g^−1^ with an increase in initial concentration from 5 to 25 mg L^−1^. This is attributed to the fact that the driving force, which depends on the concentration gradient, increases with the increasing initial Pb(II) concentration [20].

#### 2.2.2. Effect of Mass of Adsorbent

**Figure 5:**
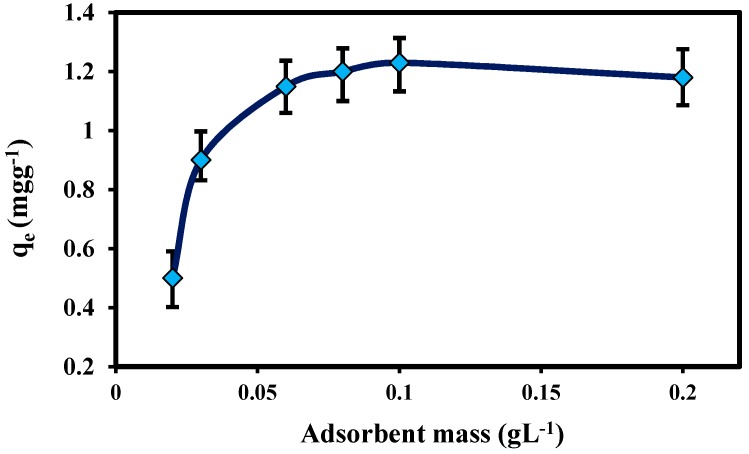
Effect of the mass of adsorbent on the Pb(II) adsorption onto ZnO-Nano-A: (solution volume, 25 mL; initial pH, 5.0; temperature, 30 °C; initial concentration, 10 mg L^−1^; contact time, 1 h).

Adsorbent dosage is an important parameter because it determines the capacity of an adsorbent for a given initial concentration of the adsorbate at the operating conditions. To determine the optimum concentration of ZnO-Nano-A, different amounts of adsorbent (0.02–0.2 g L^−1^) were added to 25.0 mL of aqueous solution containing 10.0 mg L^−1^ Pb(II) ions. Figure 5 shows that adsorption increased gradually with increasing adsorbent mass, to a maximum at 0.1 g L^−1^ for Pb(II). After this maximum equilibrium value, the adsorption capacity did not increase with increasing adsorbent mass. It is apparent that the adsorption capacity of metal ions increases rapidly with increase in the dose of the adsorbents due to the greater availability of the exchangeable sites or surface area and after that increasing these sites had no effect after equilibrium was reached [21].

#### 2.2.3. Effect of pH

**Figure 6:**
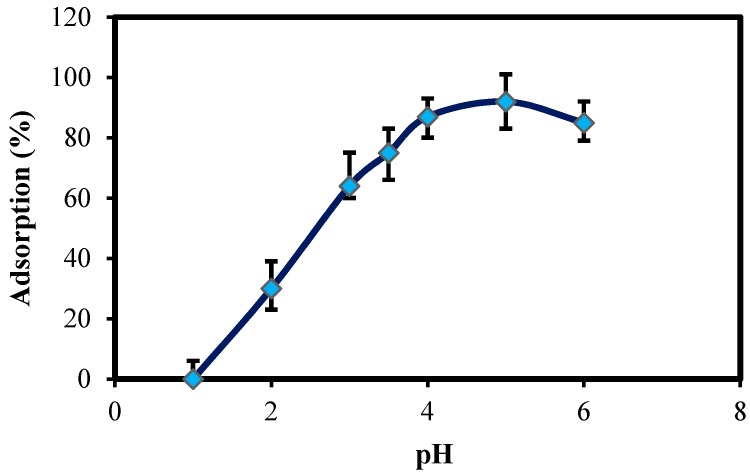
Effect of pH on the Pb(II) adsorption onto ZnO-Nano-A: (initial concentrations of Pb(II), 10 mg L^−1^; adsorbent mass, 0.1 g L^−1^; solution volume, 25 mL; temperature, 30 °C; contact time, 1 h).

The pH of the aqueous solution has been identified as the most important parameter that controls the adsorption process. The adsorption behavior of Pb(II) onto ZnO-Nano-A has been investigated at different pHs ranging from 2.0 to 6.0. Figure 6 shows that the metal uptake increased with the increase of solution pH. The maximum equilibrium uptake for Pb(II) ions was at pH 5.0, while at pH 2.0 the adsorption capacity was much lower, because large quantities of protons compete with the nano-adsorbent and decrease the absorption of metal cations [22]. Depending on the solution pH, the surface of nano-adsorbent can undergo protonation. As the pH of solution increases, the number of protons on the surface of nano-adsorbent decreases and thus more negative groups for complexation of metal cations are provided. From these results, the divalent Pb were bound to active surfaces on the lone pair electrons of oxygen from ZnO as well, C–O^−^ functional groups attached on its surface after synthesis with zerumbone, which mainly acts as active site for the complex formation with Pb ions [23].

### 2.3. Metal Adsorption Characteristics

#### 2.3.1. Adsorption Kinetics Modeling Study

Kinetics and equilibrium of adsorption are the two major parameters to evaluate adsorption dynamics. The kinetic constants of Pb(II) ions adsorption, which could be used to optimize the residence time of an industrial wastewater treated with ZnO-Nano-A, were computed using the experimental data. The adsorption equilibrium was reached with a minimum solid-solution contact time of approximately 60 min. At equilibrium time, the Pb(II) adsorption onto nano-adsorbent at pH 5.0 was found to be 93%. The two kinds of kinetic models used in this study are pseudo-first-order and pseudo-second-order equations. Lagergren-first-order equation is the most popular kinetics equation [22]. The Equation is expressed as:

(1) $$\ln\left(q_{e}-q_{t}\right)={\mathrm{lnq}}_{e}-k_{1}t$$

where q_e_ and q_t_ (mg g^−1^) are the amounts of adsorption at equilibrium and time t (min), respectively and k_1_ (min^−1^) is the rate constant of pseudo-first order adsorption.

The adsorption rate constant and k_1_ can be determined experimentally by plotting of ln(q_e_ − q_t_) against t. A pseudo second-order kinetic model [24] was used to fit the adsorption kinetic data using the following Equation:

(2) $$\frac{t}{q_{t}}=\frac{1}{k_{2}q_{e}^{2}}+\frac{t}{q_{e}}$$

where k_2_ is the rate constant of the pseudo second-order model (g mg^−1^ min^−1^).

Kinetic parameters of these models for different concentrations of lead can be determined experimentally from the slope and intercepts of the linear plots of ln(q_e_ − q_t_) against t and t/q_t_ versus t, respectively and are shown in Table 1. The rate law for a pseudo second-order kinetic model best described the experimental data with the higher correlation coefficients (R_2_). Also the calculated values of q_e_ estimated from the pseudo second-order kinetic model is much closer to the experimental values of q_e_ than that of pseudo-first-order model (Table 1). Consequently, the adsorption of Pb(II) by ZnO-Nano-A in this study was better fitted to the pseudo-second-order model.

#### 2.3.2. Adsorption Isotherm Modeling

An analysis of the relationship between adsorption capacity of nano-adsorbent and metal ion concentration was performed using the Langmuir adsorption equations [23] as:

(3) $$\frac{C_{e}}{q_{e}}=\frac{1}{{\mathrm{bQ}}_{m}}+\frac{C_{e}}{Q_{m}}$$

and the Freundlich adsorption equation [23] as:

(4) $${\mathrm{lnq}}_{e}={\mathrm{lnK}}_{F}+\frac{1}{n}{\mathrm{lnC}}_{e}$$

**Figure 7:**
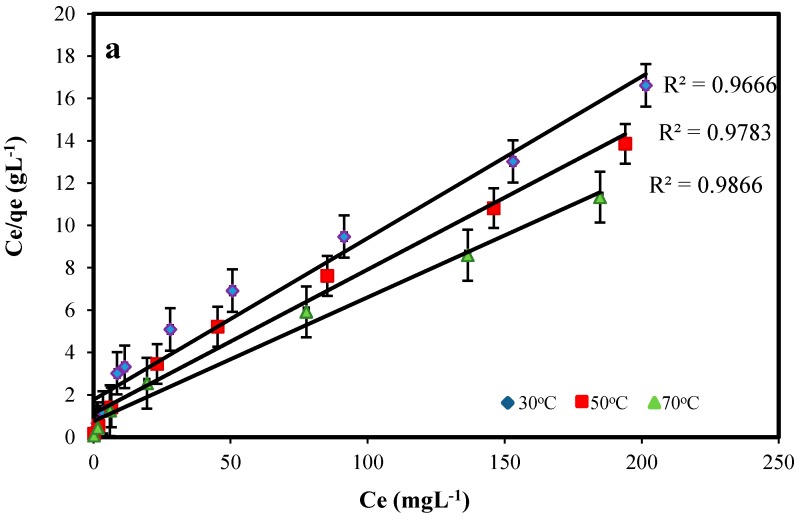

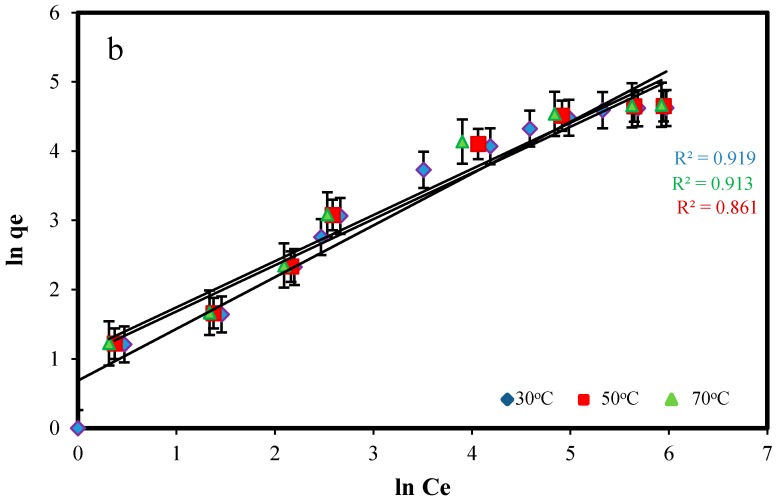
Linearized Langmuir (**a**) and Freundlich (**b**) isotherms for Pb(II) adsorption onto ZnO-Nano-A at various temperatures: contact time, 1 h; initial pH, 5.0; adsorbent mass: 0.1 g L^−1^.

The Langmuir model predicts the formation of an adsorbed solute monolayer and the Freundlich model considers the existence of a multilayered structure. The Langmuir and Freundlich isotherm parameters were calculated from the slope and intercept of linear plots of C_e_/q_e_ versus C_e_ and lnq_e_ versus lnC_e_ (Figure 7), and are given in Table 2.

From the significant correlation coefficients, the Langmuir equation was used to describe the adsorption isotherms, which fit the adsorption data better than the Freundlich equation for Pb(II) adsorption. Similar to the adsorption kinetics, the maximum equilibrium adsorption capacity (Q_m_) was obtained 19.65 mg g^−1^ for Pb(II). Since the value of n is greater than 1, it indicates favorable adsorption of metal ions on the surface of adsorbent [25]. This result is comparable with previous studies which removed lead ions from aqueous solutions using NiFe_2_O_4_ nanoparticles with 99% efficiency during 1 h [26], CuO nanoparticles with maximum adsorption capacity 37.027 mg g^−1^ after 3 h [27] and diatomite nanoparticles with maximum adsorption capacity of 103.1 mg g^−1^ at equilibrium time of 90 min [28].

#### 2.3.3. Thermodynamic Study

**Figure 8:**
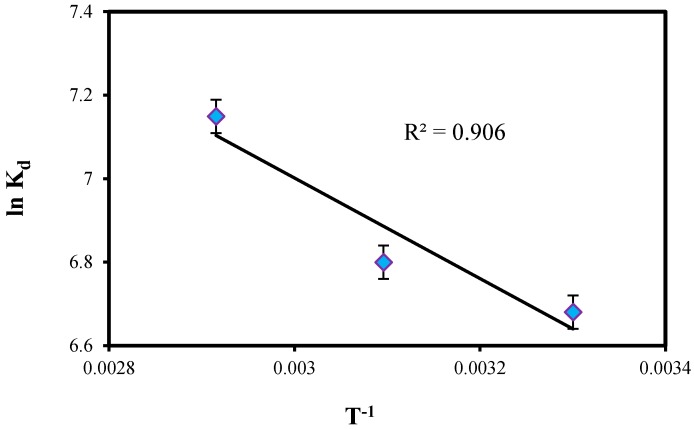
Plot of lnK_d_ vs. T^−1^ for the adsorption of Pb(II) onto ZnO-Nano-A.

The effect of temperature on the adsorption characteristics of Pb(II) was investigated by determining the adsorption isotherms at 30, 50, and 70 °C to obtain the thermodynamic parameters, which were evaluated using the Van’t Hoff equation:

(5) $$\ln K_{d}=\frac{\Delta S^{0}}{R}+\frac{\Delta H^{0}}{\mathrm{RT}}$$

where R is the gas constant (8.314 Jmol K^−1^), T the absolute temperature (K) and K_d_ is an equilibrium constant obtained by multiplying Langmuir constants Qm and b (L mol^−1^). The value of the change in enthalpy (ΔH^0^) and entropy change (ΔS^0^) during the binding process was determined from the gradient of the plots between lnK_d_ versus T^−1^. The plot shown in Figure 8 for Pb(II) was linear at the range of investigated temperatures according to Van’t Hoff equation. 

The Gibbs free energy change (ΔG^0^) of the adsorption process is related to the equilibrium constant by the classical Van’t Hoff equation:

(6) $$\Delta G^{0}=-\mathrm{RT}\ln K_{d}$$

The calculated thermodynamic parameters such as ΔH^0^, ΔS^0^ and ΔG^0^ for the adsorption system are given in Table 3. The adsorption of lead ions has been found to increase with an increase in temperature from 30 to 70 °C. It can be seen from Table 3 that ΔH^0^ values obtained was positive and also observed that the distribution coefficient values, K_d_ increased with increase in temperature which shows the endothermic nature of the adsorption. The positive value of ΔS^0^ suggested the increasing randomness at the solid/liquid interface during the adsorption of Pb(II) ions on ZnO-Nano-A. The negative values of ΔG^0^ indicated that the adsorption was spontaneous and the decrease in the value of ΔG^0^ with increasing temperature shows that the reaction was favorable at higher temperature [29].

## 3. Materials and Methods

### 3.1. Materials

Pb(NO_3_)_2_ was purchased from Fluka (Morris Plains, NJ, USA). All other chemicals were of analytical reagent grade and were used without further purification. *Zingiber zerumbet* rhizome was obtained from a local market in Kuala Lumpur, Malaysia.

### 3.2. Extraction of Zerumbone

Zerumbone crystals were prepared according to process described in previous study [30] and yielded 1.45 g/kg rhizome. Briefly, Fresh *Zingiber zerumbet* rhizome (1 kg) was washed several times with water, cut to small pieces and boiled with deionized water in a distillation hydration apparatus to obtain the essential oil. The oil was crystallized using absolute 100% *n*-hexane (Sigma-Aldrich, Kuala Lumpur, Malaysia) and the solution was then left to evaporate in a fume hood (Novaire, Newton, MA, USA). Recrystallization was carried out three times to achieve pure zerumbone crystals. The purity of zerumbone was determined using a high performance liquid chromatography (HPLC) system (Waters, Milford, MA, USA) was 99.97%. Pure zerumbone crystals were collected in clean glass bottles and kept at 4 °C.

### 3.3. Synthesis of ZnO NPs 

Zerumbone crystals (about 1 g) was dissolved in ethanol (98%, 100 mL) under gentle stirring at room temperature. After complete dissolution, zinc acetatedehydrate (Zn(Ac)_2_·2H_2_O, 2.19 g) was added to react with the zerumbone solution for 2 h under continuous magnetic stirring at 70 °C. The white color solid product was collected by centrifugation at 8000 rpm for 15 min and washed carefully with ethanol to remove surplus zerumbone and then dried at 100 °C for 2 h.

### 3.4. Characterization of ZnO NPs

The ZnO NPs were characterized by PXRD (Philips, X’pert, Almelo, The Netherlands), at 40 kV and 30 mA from 2θ = 10° to 80° with nickel-filtered Cu (λ = 1.542 Å) at room temperature. FT-IR spectra of the samples were recorded over the range of 400–4000 cm^−1^ by a Model spectrum 100 series (Perkin Elmer, Waltham, MA, USA) FTIR spectrophotometer. UV-vis spectra of ZnO NPs powders were measured using a spectrophotometer (Lambda 25-Perkin Elmer) in wavelength between 200 and 800 nm. The morphology and size of ZnO-NP samples were examined by using a Transmission Electron Microscope (TEM, Hitachi H-700, Tokyo, Japan) in 120 kV. The particle electrostatic charge was evaluated using the laser doppler electrophoresis technique, whereby 100 µL of the solution was diluted in 1.5 mL of water. Then it was poured into a Zeta sizer-nano instrument cuvette (Malvern, UK); the results are stated as zeta potential (ZP). Concentration of Zn^2+^ ions before and after addition of extract was measured using Inductively Coupled Plasma Atomic Emission Spectroscopy (ICP-AES) model Perkin Elmer 1000. Following Equation was used to calculate percentage yield (%Y) using initial concentration (IC) and final concentration (FC) of Zn^2+^ ions:

(7) $$\%Y=\frac{\mathrm{Ic}-\mathrm{Fc}}{\mathrm{Ic}}\times 100\%$$

### 3.5. Adsorption Experiments

The adsorption of Pb(II) ions on ZnO NPs as nano-adsorbent was studied by a batch technique. The stock solution of 1000 mg L^−1^ Pb(II) was prepared by dissolving a weighed quantity of Pb(NO_3_)_2_ in distilled water. The metal solutions were prepared in distilled water by gradually diluting the stock solution to desired concentrations. The adsorption behaviors of Pb(II) ions by ZnO-Nano-A were investigated in the pH range 2.0 to 6.0 at 30 °C. The effect of several parameters such as pH, concentrations, contact time and adsorbent mass on the adsorption were studied. The initial pH of the adsorbate solution was adjusted using 1 M HCl or 1 M NaOH aqueous solution without any further adjustment during the adsorption process. Adsorption isotherm of Pb(II) on ZnO-Nano-A was carried out from batch experiments by contacting 0.1 g of the ZnO-Nano-A with 25 mL of varying concentrations of Pb(II) from 5 to 250 mg L^−1^ for 1 h on a mechanical stirrer at different temperature (30, 50 and 70 °C). The pH of the solution was adjusted to an optimum pH. After reaching adsorption equilibrium, the adsorbent was removed through centrifuge, and the concentration of metal ions remaining in absorbent was measured using ICP-AES. For Pb(II) adsorption kinetics studies, 0.1 g of ZnO-Nano-A was contacted with 25 mL of Pb(II) solution of varying concentrations in a flask and stirred continuously at different times. At the end of the pre-determined time intervals, the adsorbent was separated by centrifuge. The residual concentration of Pb(II) in the absorbent was determined using ICP-AES. The results of these studies were used to obtain the optimum conditions for maximum metal removal from aqueous solution. The amount of metal ions adsorbed onto the unit amount of the asorbents, q_e_ (mg g^−1^), was calculated using the following equation:

(8) $$q_{e}=\frac{\left(C_{0}-C_{e}\right)V}{W}$$

where C_0_ and C_e_ are initial and final metal ions concentrations (mg L^−1^), respectively, V is the volume of lead solution (L), and W is the dry mass of the adsorbent (g). The percent adsorption of metal ions was calculated as follows:

(9) $$\mathrm{Adsorption}\%=\frac{C_{0}-C_{e}}{C_{0}}\times 100$$

## 4. Conclusions

Nanoparticles provide an efficient technique for the removal of toxic heavy metals from wastewater. In this study, ZnO NPs were successfully prepared with a safe, simple and economic process using of zerumbone extract and evaluated their efficiency as novel nano-adsorbent for removal of lead ions in aqueous solution. The biosynthesized nanoparticles exhibited an excellent adsorption for the Pb(II) ions that followed Langmuir adsorption model and pseudo-second-order equation. The maximum adsorption capacity of Pb(II) was found to be 19.65 mg g^−1^ under pH of 5, and temperature of 70 °C in aqueous solution. The enhanced adsorption at higher temperature indicates endothermic adsorption process.

## Figures and Tables

**Table 1:** Kinetic parameters of Pb(II) adsorbed onto ZnO-Nano-A.

Pseudo-First Order					Pseudo-Second Order		
Conc (mg L^−1^)	q_e_ exp (mg g^−1^)	k_1_ (min^−1^)	q_e_ (cal)	R^2^	k_2_ (g mg^−1^min^−1^)	q_e_ (cal)	R^2^
10	2.1	4.8 × 10^−2^	1.3	0.868	22.7 × 10^−2^	2.2	0.999
20	3.7	2.3 × 10^−2^	4.2	0.817	10.7 × 10^−2^	3.8	0.989
30	4.8	1.4 × 10^−2^	5.8	0.959	4.8 × 10^−2^	4.5	0.997

**Table 2:** Isotherm constants of Pb(II) adsorbed onto ZnO-Nano-A.

Langmuir Isotherm				Freundlich Isotherm		
Temp (°C)	Q_m_ (mg g^−1^)	B (L mg^−1^)	R^2^	K_F_ (mg g^−1^)	n	R^2^
30	15.65	5.93 × 10^−2^	0.988	1.98	2.34	0.929
50	17.84	7.37 × 10^−2^	0.999	2.94	2.54	0.961
70	19.65	8.39 × 10^−2^	0.999	3.16	2.81	0.914

**Table 3:** Thermodynamic parameters of Pb(II) adsorbed onto ZnO-Nano-A.

Temp (K)	ΔH^0^ (kJ mol^−1^)	ΔG^0^ (kJ mol^−1^)	ΔS^0^ (J mol^−1^ K^−1^)
**30**	11.13	–16.19	0.7
**50**		−18.40	
**70**		–19.91	
